# Post‐operative injection of hydrolyzed collagen peptides shows anti‐inflammatory effect in patients with femoroacetabular impingement improving the early recovery

**DOI:** 10.1002/jeo2.70158

**Published:** 2025-01-31

**Authors:** Enrico Tassinari, Andrea Minerba, Tommaso Basile, Alessio Bucciarelli, Francesco Traina, Brunella Grigolo, Stefano Zaffagnini, Eleonora Olivotto

**Affiliations:** ^1^ 2nd Orthopaedic and Traumatologic Clinic IRCCS Istituto Ortopedico Rizzoli Bologna Italy; ^2^ Orthopaedic‐Traumatology and Prosthetic Surgery and Revisions of Hip and Knee IRCCS Istituto Ortopedico Rizzoli Bologna Italy; ^3^ RAMSES Laboratory, RIT Department, Research Centre Codivilla‐Putti IRCCS Istituto Ortopedico Rizzoli Bologna Italy

**Keywords:** arthroscopy, cortisone, femoroacetabular impingement, hydrolyzed collagen peptides, osteoarthritis

## Abstract

**Purpose:**

This study aimed to compare the use of cortisone (C), intra‐articular injected at the end of hip arthroscopy in patients with femoroacetabular impingement (FAI), to a new Class III medical device based on hydrolyzed collagen peptides ‘PEPTYS’ (P) and, to investigate potential associations among preoperative symptoms and hip function, outcomes after arthroscopic surgery and presence of inflammatory biomarkers in synovial fluids (SFs) at basal condition.

**Methods:**

The two treatments were administrated to patients scheduled for arthroscopy with simple blind randomization sampling. Based on the sample size calculation, the number necessary to recruit was at least 20 patients for the C group and 20 for the P group. SFs, when available, were obtained by aspiration just prior to surgical intervention. At the baseline, osteoarthritis (OA) severity was assessed with a radiographic scoring system (Tönnis classification). Physical examination and clinical assessment using the Hip disability and Osteoarthritis Outcome Score (HOOS) and visual analogue scale (VAS) score for pain were performed at the time of surgery and at 1 and 6 months of follow‐up. At the time of surgery, chondral (Outerbridge score) and labral pathology based on direct arthroscopic visualization were also evaluated.

**Results:**

Forty‐seven FAI patients were enroled, with a median age of 35 years with a standard deviation (SD) of 10.6 and a body mass index of 24.3kg/m² with an SD of 4.5. 24 patients were treated with C and 23 with P. Both treatments did not show any statistically significant difference in hip function and pain. High expression of inflammatory molecules in SFs was correlated with the worst post‐operative articular function.

**Conclusions:**

Our study showed that the use of P was completely comparable to cortisone. Therefore, PEPTYS might be a valuable candidate to improve early recovery, in terms of pain and function, from arthroscopic FAI treatment.

**Level of Evidence:**

Level III, comparative and randomized study.

**Abbreviations**
BMAC: bone marrow aspirate concentrate
BMI: body mass index
C: cortisone
CCL: chemokine (C‐C motif) ligand
COMP: Cartilage Oligomeric Matrix Protein
CTX‐II: C‐telopeptide fragments of type II collagen
ELISA: enzyme‐linked assay
FAI: femoroacetabular impingement
funct: function
HOA: hip osteoarthritis
HOOS: Hip disability and Osteoarthritis Outcome Score
IL: interleukin
JSN: joint space narrowing
MCID: minimal clinically important difference
MCP‐1: monocyte chemoattractant protein‐1
MD: medical device
MIB‐3‐b: macrophage inflammatory protein‐3‐beta
OA: osteoarthritis
P: PEPTYS
PRP: platelets‐rich plasma
qol: quality of life
SD: standard deviation
SF: synovial fluid
sym: symptoms
tot: total
VAS: visual analogue scale
VEGF: vascular endothelial growth factor

## INTRODUCTION

Femoroacetabular impingement (FAI) is a set of anatomical alterations of the proximal femur (Cam) and/or the acetabulum (Pincer) that cause an anomalous contact between the acetabular rim and the head‐neck junction of the femur [11]. These anatomical alterations can produce synovial inflammation, damage to the cartilage, labrum and surrounding capsular structures, leading to pain, loss of function and potentially early hip osteoarthritis (HOA) especially in young adults [19]. Recent evidence from prospective cohort studies has demonstrated that FAI is involved in the early development of HOA [2], which is often associated with synovial inflammation correlated with joint pain, dysfunction, and more rapid progression of HOA itself [4, 12, 21, 24]. Surgical treatment of FAI should be consequent in case of negative response to conservative treatments and should restore the normal anatomy of the hip, through a complete correction of the deformities that led to the chondrolabral damage. Open dislocation, mini‐open, and arthroscopic surgery are effective methods for symptomatic FAI treatment as shown in short‐ and midterm studies [16].

Hip arthroscopy is a minimally invasive surgical procedure, under ordinary hospitalization or day‐hospital). To improve early recovery from arthroscopic surgery, at the end of the procedure, several substances could be injected inside the joint, but there is no consensus about which one is better. Literature is lacking studies concerning this specific topic [13].

In our department, we usually inject cortisone with local anaesthetic; the rationale is to combine the anti‐inflammatory effect of cortisone with the analgesic effect of the anaesthetic.

Hydrolyzed collagen peptides have recently been described in treating OA, administered by intra‐articular injection, with good results. They seem to reduce pain and improve function [9, 28]. Their effect has not been studied yet in the intra‐operative setting. At our best effort, we did not find literature studies concerning the administration of collagen peptides at the end of hip or knee arthroscopy.

The purpose of this study was to compare in terms of post‐operative pain relief and hip functional improvement the use of cortisone plus anaesthetic administered intra‐articular (1 cc of DEPO‐MEDROL 40 mg/mL + 1 cc of NAROPINA 0.75% 7.5 mg/mL) at the end of arthroscopic procedure, with a new Class III medical device (MD)—PEP‐52, PEPTYS (5 mg/mL, 2 mL) based on hydrolyzed collagen peptides. Moreover, we investigate potential associations among preoperative symptoms and hip function, outcomes after arthroscopic surgery and biomarkers in synovial fluids (SFs).

We hypothesize that PEPTYS might be suggested as an alternative option to cortisone in post‐operative hip arthroscopy injections, showing better or at least the same clinical results without harmful effects on joint cartilage.

## METHODS

### Study design

This is a comparative and randomized study including consecutive patients undergoing hip arthroscopy for FAI. The study was conducted in accordance with the Declaration of Helsinki, and the protocol was approved by the Local Ethical Committee (Prot. gen. 0008219) and registered on clinicaltrials.gov. The affected hip is placed in gradual traction to increase the joint space enough to introduce the arthroscopic instruments. The acetabular labrum is repaired, and the CAM deformity is corrected. To improve the outcome of the arthroscopy, at the end of the arthroscopic procedure, cortisone was injected for its anti‐inflammatory properties. In the present study patients were alternatively injected with the new MD (PEPTYS).

One orthopaedic surgeon with more than 10 years of experience in hip arthroscopy [E.T.] carried out all procedures following standard approaches [5, 25]. The choice between cortisone and the new medical device took place before surgery, with randomized computer‐based methods and with blinded patients.

### Medical device

The new medical device PEPTYS (ref. code: PEP‐52, Tiss'You Srl, RSM) is a solution of low molecular weight peptides derived from hydrolyzed collagen of bovine origin with vitamin C (stabilized ascorbic acid) and phosphate‐buffered saline and it will be used according to the indication for which the CE marking was obtained (i.e., intra‐articular infiltration in single administration). The MD concentration was 5 mg/mL in a 2 mL single administration.

### Patient recruitment and clinical data

Patients were enroled after providing written informed consent and if eligibility criteria were met. Inclusion criteria were age 18–65 years and clinical diagnosis of symptomatic FAI. Exclusion criteria were listed as follows: inability to provide informed consent; dysplastic hips (lateral centre‐edge angle: <20°), malignancies and overall poor general condition of health; history of tumour or infection; established diagnosis of rheumatic diseases; arthroscopic surgery performed for any other reason than FAI and/or labral pathology; previous operations at the affected hip or clinical and radiographic signs of HOA (Tönnis classification >2); history of diabetes, and neurologic disease; pregnant or breastfeeding women; patients with proven hypersensitivity to collagen of bovine origin or vitamin C.

The study obtained extensive records of demographics (age, sex, body mass index [BMI]), pre‐operative preclinical and post‐operative data from patients. To assess patients' opinions about their hip and associated problems, and to evaluate their symptoms and functional limitations, all patients completed a Hip disability and Osteoarthritis Outcome Score (HOOS), validated in Italian language (http://www.koos.nu/hoositalian.pdf) at the time of surgery, at 1 and at 6 months of follow‐up.

A visual analogue scale (VAS) was used to measure pain intensity providing a range of scores from 0 to 10. A higher score indicates greater pain intensity [10]. It was obtained at the time of surgery, at 1 and at 6 months of follow‐up.

Plain anteroposterior radiographs in standing position were obtained before surgery to evaluate the progressive degrees of degenerative changes to the hip using the Tönnis scale [26]. Briefly, a Grade 0 means a hip absent of arthrosis. Grade 1 = slight joint space narrowing (JSN), slight lipping at the joint margin, and slight sclerosis; Grade 2 = presence of small bony cysts, further JSN, and moderate loss of femoral head sphericity; Grade 3 = most severe indicates large cysts, severe JSN, severe femoral head deformity, and avascular necrosis. Patients were followed by visits at 1 and 6 months after surgery, to assess the anti‐inflammatory effect of the treatment at early post‐operative outcomes without the use of any other drug. Interestingly, previous studies have demonstrated that 6‐month can also be predictive of long‐term outcomes [3].

### Macroscopic findings and sample collection

Macroscopic intra‐articular pathology was graded using the Outerbridge score for chondral lesions [20].

At basal condition, SF was collected from patients by aspiration before arthroscopy and centrifuged, then aliquoted and stored at −80°C until analysis.

Also, urine samples were also collected and stored frozen at −80°C for longer storage terms. Prior to use, urine samples were thawed gradually at room temperature and centrifuged before the analysis.

### Biomarkers analysis

Inflammatory biomarkers as cytokines (interleukin‐6 [IL‐6], IL‐1β, transforming growth factor α) and chemokines (IL‐8, chemokine [C‐C motif] ligand [CCL]2/MCP‐1, CCL5/RANTES, CCL19/MIP‐3‐b and CCL21/6kine), typically involved in OA pathology, were simultaneously evaluated in the SFs at basal condition with commercially available multiplex bead‐based sandwich immunoassay kits (Bio‐Rad Laboratories). Data were analyzed by using the Bio‐Plex Manager software version 6.0 (Bio‐Rad Laboratories).

Vascular endothelial growth factor (VEGF), a potent mediator of both angiogenesis and vasculogenesis, a marker of inflammation, was also detected in SFs with a sandwich enzyme‐linked assay (ELISA) Kit (Human VEGF Quantikine ELISA Kit, R&D Systems, Inc.).

Two sensitive markers of cartilage degradation in OA, C‐telopeptide fragments of type II collagen (CTX‐II) and Cartilage Oligomeric Matrix Protein (COMP), were evaluated in SFs and urine using commercially available Sandwich‐ELISA Kits (Elabscience Biotechnology Inc.). The kit standard curves were fit with a four parameters logistic curve, based on these curves, the unknown concentrations were calculated starting from the optical densities red with a microplate reader Infinite® M Plex (Tecan Trading AG) (the equations used are described in Supporting Information S1: Table 1).

### Statistical analysis

For the sample size calculation of 40 patients (20 cortisone group and 20 peptide group), we assumed from the literature [15] that the pain reduction (VAS) in the group treated with cortisone was 2 points at 1 month (T0) (standard deviation [SD] = 3.3), and that in the group treated with peptides the average reduction in pain 5 points. The sample size was set to carry out a *t* test comparing the mean of the VAS between the two groups, with a significance level (*α*) of 0.05 and a power of 80%.

A preliminary analysis of the collected data was done using Spearman or Pearson's correlation matrix, depending on the type of data. Binary categorical variables were treated as numerical to understand their potential impact on the HOOS and VAS score. Values were assigned as follows: 0 for cortisone treatment, female sex, left side, no labrum suture/shaving or microfracture and no acetabuloplasty or femoroplasty; 1 for PEPTYS treatment, male sex, right side, labrum suture/shaving, microfracture and acetabuloplasty or femoroplasty. To correct baseline differences in HOOS and VAS score between cortisone and PEPTYS treatments, the mean difference between groups pre‐intervention was calculated and added to the group with the lower pre‐intervention mean at each time point. The results were then normalized to a range of [0, 100] using feature‐scaling (as shown in Supporting Information S1: Table 1). This approach removed pre‐existing differences between patients, enabling a direct easy comparison between the two groups. The same method was applied to VAS scores. To assess statistical differences between cortisone and PEPTYS treatments, a two‐way ANOVA was performed, followed by Šídák's multiple comparisons test, with significance levels: *p* ≤ 0.05 (*), *p* ≤ 0.01 (**), *p* ≤ 0.001 (***) and *p* ≤ 0.0001 (****).

For evaluating treatment efficacy, ‘responders’ were identified based on the minimal clinically important difference (MCID) using a distribution‐based approach. MCID was calculated as 0.5 times the baseline score's SD. Baseline (Month 0) and final (Month 6) scores for each patient were compared across HOOS subscales and the VAS score for pain. The HOOS subscales analyzed included total score, symptoms, pain, daily activities, sports and recreation and quality of life. MCID values were determined for each HOOS subscale and the VAS score at baseline. Patients were considered ‘responders’ if their final scores exceeded the baseline by at least the MCID for HOOS subscales or if their VAS score decreased by at least the MCID. The percentage of responders was then calculated for each treatment group (cortisone and PEPTYS). Chi‐square tests, or Fisher's exact test when expected counts were less than five, were used to compare the number of responders between the treatment groups for each outcome measure, with p‐values reported and significance set at *p* < 0.05.

A multivariate linear regression was performed on each HOOS subscale to assess factors including treatment, sex, age, BMI, affected side, and other clinical characteristics such as chondropathy, Tonnis grading, labrum treatment, microfractures, and time points. The regression used robust standard errors (HC3) [27]. All the analyses were performed using the R programming language (v.4.3.1) and the R studio IDE (v.2023.06.1).

## RESULTS

### Patient demographics and baseline characteristics

**Figure 1:**
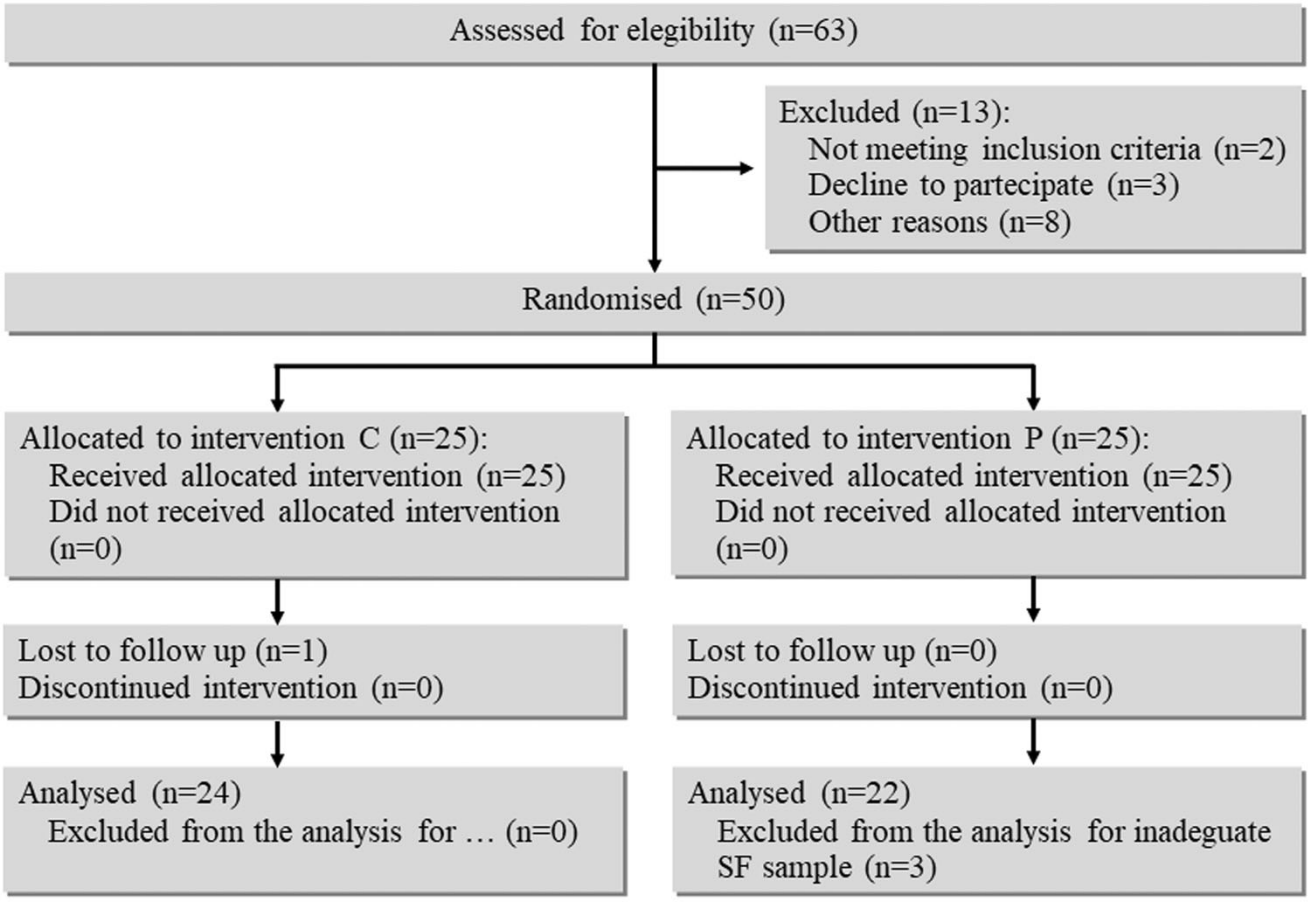
Flow diagram of FAI patients included in the study. FAI, femoroacetabular impingement.

Out of 63 consecutive patients undergoing arthroscopy for FAI enroled in the study, 17 patients were excluded. Finally, 46 SF samples were available for analysis. A flow diagram of the progress through the phases of a parallel randomized trial of two groups (enrolment, intervention allocation, follow‐up, and data analysis), following the Consolidated Standards of Reporting Trials (CONSORT) guidelines [23], is represented in Figure 1. These patients had an initial diagnosis of cam FAI in 24 cases, pincer FAI in 4 cases and mixed FAI in the remaining cases.

Frayed labral tissue in the impingement area was sutured in about 60% of patients and 34% was shaved. Few patients underwent microfractures and about 90% of them had femoroplasty, while 30% had acetabuloplasty. Patient demographics and clinical features at baseline are summarized in Table 1; hip functionality and pain at the baseline, measured respectively with the HOOS and VAS score, are described in Table 2. During the surgical procedure, 25 patients were injected with cortisone and 25 with PEPTYS.

**Table 1:** Descriptive statistics of the patient population at baseline (47 patients).

Variable	Unit	Cortisone	Peptides
Male/female	Number (%)	11 (45.83)/13 (54.17)	16 (69.56)/7 (30.43)
Age (mean ± SD)	Years	37.00 ± 11.15	33.30 ± 10.24
BMI (mean ± SD)	Kg/m^2^	24.42 ± 5.02	24.23 ± 4.18
Right/left hip	Number (%)	10 (41.67)/14 (58.33)	15 (65.22)/8 (34.78)
Acetabular chondropathy (yes/no)	Number (%)	23 (95.83)/1 (4.17)	19 (82.61)/4 (17.39)
Femoral chondropathy (yes/no)	Number (%)	18 (75.00)/6 (25.00)	13 (56.52)/10 (43.48)
TöNNIS scale (0–2)	Scale value, number (%)	0, 10 (41.67) 1, 12 (50.00) 2, 2 (8.33)	0, 15 (68.18) 1, 7 (31.81) 2, 0 (0.00)
Labrum suture (yes/no)	Number (%)	14 (58.33)/10 (41.67)	15 (65.27)/8 (34.78)
Labrum shaving (yes/no)	Number (%)	9 (37.50)/15 (62.50)	7 (30.43)/16 (69.56)
Microfractures (yes/no)	Number (%)	3 (12.50)/21 (87.50)	1 (4.35)/22 (95.65)
Acetabuloplasty (yes/no)	Number (%)	10 (41.67)/14 (58.33)	6 (26.10)/17 (73.91)
Femoroplasty (yes/no)	Number (%)	20 (83.33)/4 (16.67)	23 (100.00)/0 (0.00)

*Note*: Data are expressed in terms of mean ± SD, being the populations normally distributed.

Abbreviations: BMI, body max index; SD, standard deviation.

**Table 2:** Descriptive statistics of the HOOS and VAS scoring at the baseline.

Variable	Unit	Cortisone	Peptide
HOOS total	%	45.00 ± 22.50	57.50 ± 28.50
HOOS symptom	%	45.00 ± 26.25	60.00 ± 22.50
HOOS pain	%	40.00 ± 28.13	57.50 ± 32.50
HOOS functional	%	53.65 ± 26.45	67.60 ± 38.20
HOOS sport	%	34.40 ± 26.55	50.00 ± 37.50
HOOS quality	%	21.90 ± 17.18	37.50 ± 18.80
VAS		7.00 ± 4.00	5.00 ± 6.00

*Note*: Data are expressed in terms of median ± IQR.

Abbreviations: HOOS, Hip disability and Osteoarthritis Outcome score; IQR, interquartile range; VAS, visual analogue scale.

### Clinical outcome of FAI patients after arthroscopy

A Spearman correlation matrix was performed as an explorative data analysis with both clinical and biological data to evaluate the presence of correlations (Supporting Information S1: Figure 1). In the correlation matrix, both the HOOS and VAS score were reported as bare values and as normalized values, as explained in the previous specific section. All the information present in the bare HOOS and VAS score was conserved by the normalization as proven by the strong direct correlation.

**Figure 2:**
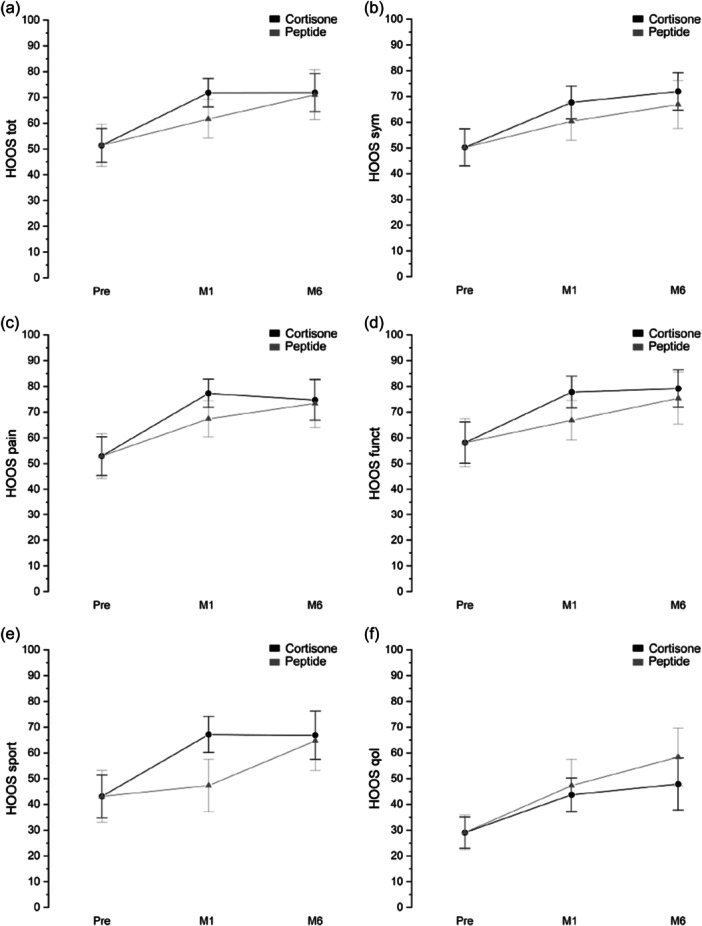
HOOS interval plot (mean ± 95% CI) at different time points (basal, after 1 and 6 months) of the two treatment groups (cortisone, peptides) corrected considering the difference among the mean pre‐treatments. (a) Total score. (b) Symptoms. (c) Pain. (d) Function in daily living. (e) Function in sport and recreative activities. (f) Quality of life. CI, confidence interval; HOOS, Hip disability and Osteoarthritis Outcome Score; M1–6, months 1–6.

Concerning the clinical outcomes, the ‘treatment’ results to be not influential on the HOOS and VAS, meaning that the two treatments may be considered as interchangeable. From an initial analysis, HOOS and VAS score were slightly correlated to the time point; the correlation is slightly negative, and this is due to the presence of few outliers (Supporting Information S1: Figure 2).

The two treatments resulted to be equally performant in terms of HOOS and VAS score, with a trend along the months with an increase in the score due to the recovery. Interestingly the HOOS was almost always lower, at Month 1, for the peptide treatment. However, at Month 6, the HOOS of the two treatments was almost identical (Figure 2).

**Figure 3:**
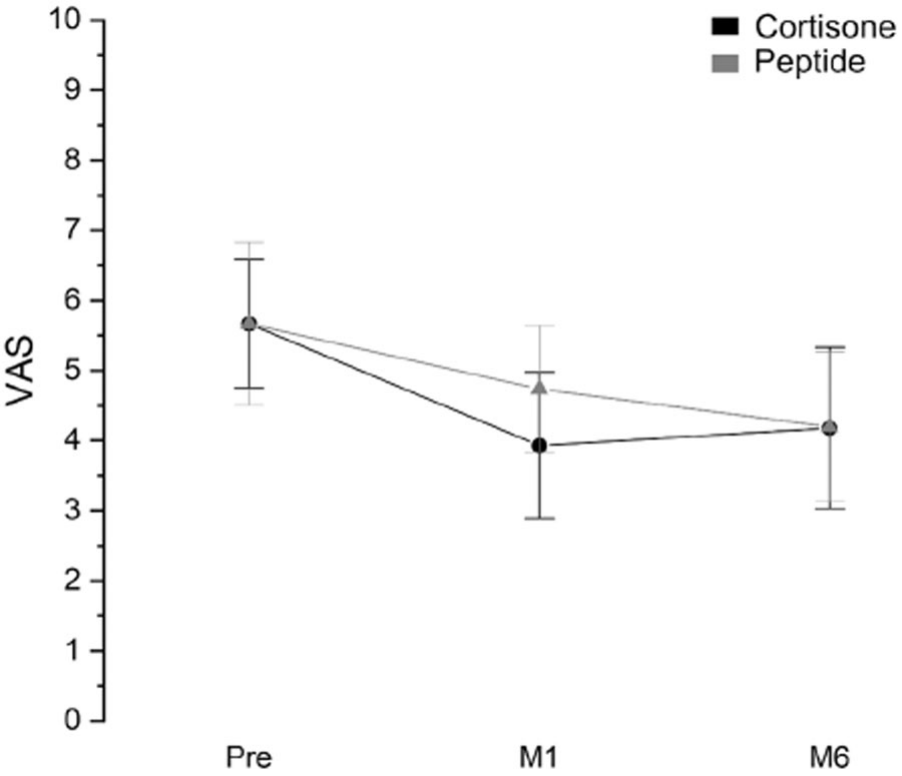
VAS interval plot (mean ± 95% CI) at different time points (pre‐treatment, after 1 month, and after 6 months) of the two treatment groups (cortisone, peptides corrected considering the difference among the mean pre‐treatment). CI, confidence interval; M1–6, months 1–6; VAS, visual analogue scale.

Also, as for the VAS score there was no statistical difference between the two treatments (Supporting Information S1: Tables 2–8). The trend was, as expected, inverted with respect to HOOS: a lower score implies a lower pain; thus, a decrease in VAS indicates a recovery (Figure 3).

Noteworthy, based on the MCID analysis of the HOOS subscales and VAS score, PEPTYS treatment demonstrates comparable efficacy to cortisone across all evaluated outcome measures. The percentage of responders, defined as patients whose final score improved by at least 0.5 SD from baseline, shows little variation between the two treatment groups, indicating that PEPTYS performs similarly to cortisone in managing patient outcomes after surgery (Table 3).

**Table 3:** MCID analysis on the different scores, summary of number of non‐responders and responders, percentage of responders for each treatment group and *p* values of two statistical tests (chi‐squared and Fisher).

Score	MCID	Cort Res	Cort Non‐Res	Pept Res	Pept Non‐Res	Res‐Cort (%)	Res‐Pept (%)	*p* (*χ* ^2^)	*p* (Fish.)
HOOS tot	10.06	17	6	16	7	73.91	69.57	1.00	1.00
HOOS sym	9.70	11	12	14	9	47.83	60.87	0.55	0.55
HOOS pain	11.15	16	7	16	7	69.57	69.57	1.00	1.00
HOOS funct	11.54	16	7	14	9	69.57	60.87	0.76	0.76
HOOS sport	12.25	16	7	17	6	69.57	73.91	1.00	1.00
HOOS qol	8.82	12	11	15	8	52.17	65.22	0.64	0.65
VAS	1.35	9	14	10	13	39.13	43.48	0.90	0.90

*Note*: Baseline (Month 0) and final (Month 6) scores for each patient were compared across HOOS subscales and the VAS score.

Abbreviations: HOOS, Hip disability and Osteoarthritis Outcome Score; MCID, minimal clinically important difference; Non‐Res, non‐responders; Res, responders; VAS, visual analogue scale.

To understand the impact of the clinical factors on the HOOS, a general linear regression was used. The model was performed to fit only the main effects of the clinical data from the patients (age, BMI, time to surgery [0], treatment [0], sex [0], acetabular chondropathy ≥2, femoral chondropathy ≥2, labrum suture [0], Tönnis ≥1, labrum shaving [0], acetabularplasty [0], femoroplasty [0] and microfractures [0]). All the categorical variables were considered in the 0 state (Table 1), and the coefficient for the other state (1) was set to 0 to avoid redundancy. The coefficients of the general linear regression are reported in Supporting Information S1: Table 9 within the significance and 95% low and high confidence intervals.

The total HOOS was significantly worse in older patients and in patients with higher BMI (*p* = 0.049 and 0.022, respectively) as the HOOS subscales function (*p* = 0.029 and 0.021, respectively) and sport (*p* = 0.041 and 0.006, respectively). Moreover, the HOOS pain was also significantly worse in patients with higher BMI (p = 0.015). The absence of microfracture showed significantly better HOOS symptoms, sport and quality of life (*p* = 0.013, *p* = 0.010 and *p* = 0.013). Related to the surgical correction of abnormal joint structure, the absence of CAM resection was significantly correlated to better HOOS symptoms, quality of life and pain (*p* = 0.005, *p* = 0.038 and *p* = 0.041).

The same analysis was applied to the VAS score showing any statistically significant correlation with clinical data (Supporting Information S1: Table 10).

### Biomarkers analysis and correlation with clinical data

In general, the concentration of the selected cytokines in the SFs of FAI patients, collected at the baseline, was lower compared to the chemokines (Supporting Information S1: Figure 3).

**Figure 4:**
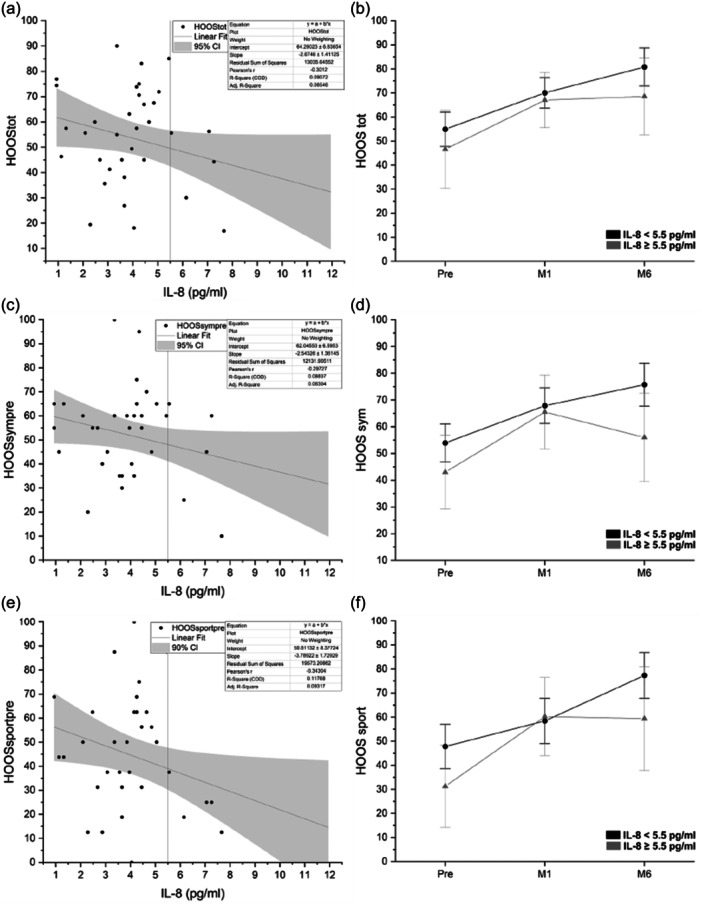
Scatter plots of IL‐8 versus the HOOS subscales at the baseline (pretreatment) and the effect of the over time of the chosen threshold. (a) Scatter plot of HOOS tot at the baseline versus IL‐8, (b) HOOS tot over time divided by the threshold. (c) Scatter plot of HOOS sym at the baseline versus IL‐8, (d) HOOS sym over time divided by the threshold. (e) Scatter plot of HOOS sport at the baseline versus IL‐8, (f) HOOS sport over time divided by the threshold. HOOS, Hip disability and Osteoarthritis Outcome Score; M1–6, Months 1–6.

The correlations among the biological markers and the HOOS and VAS score were also explored in detail (Pearson's coefficient ‐0.3 ≥ *r* ≥ 0.3) (Supporting Information S1: Figure 4).

Among the cytokines and chemokines analyzed, the most influential marker was IL‐8, which resulted in an inverse correlation with total HOOS (*r* = −0.3), HOOS symptoms (*r* = 0.3) and HOOS sport (*r* = −0.34). The scatter plot with the linear regression of these HOOS subscales versus IL‐8 is reported in Figure 4a,c,e, respectively. We set a threshold at IL‐8 = 5.5 pg/mL because, in all three subscales, above this value, no patient with an HOOS >65 was recorded. Patients with an IL‐8 ≥5.5 pg/mL tend to have lower scores, as reported in Figure 4b (HOOS tot), Figure 4d (HOOS sym) and Figure 4F (HOOS sport), even if the differences were not statistically significant.

**Figure 5:**
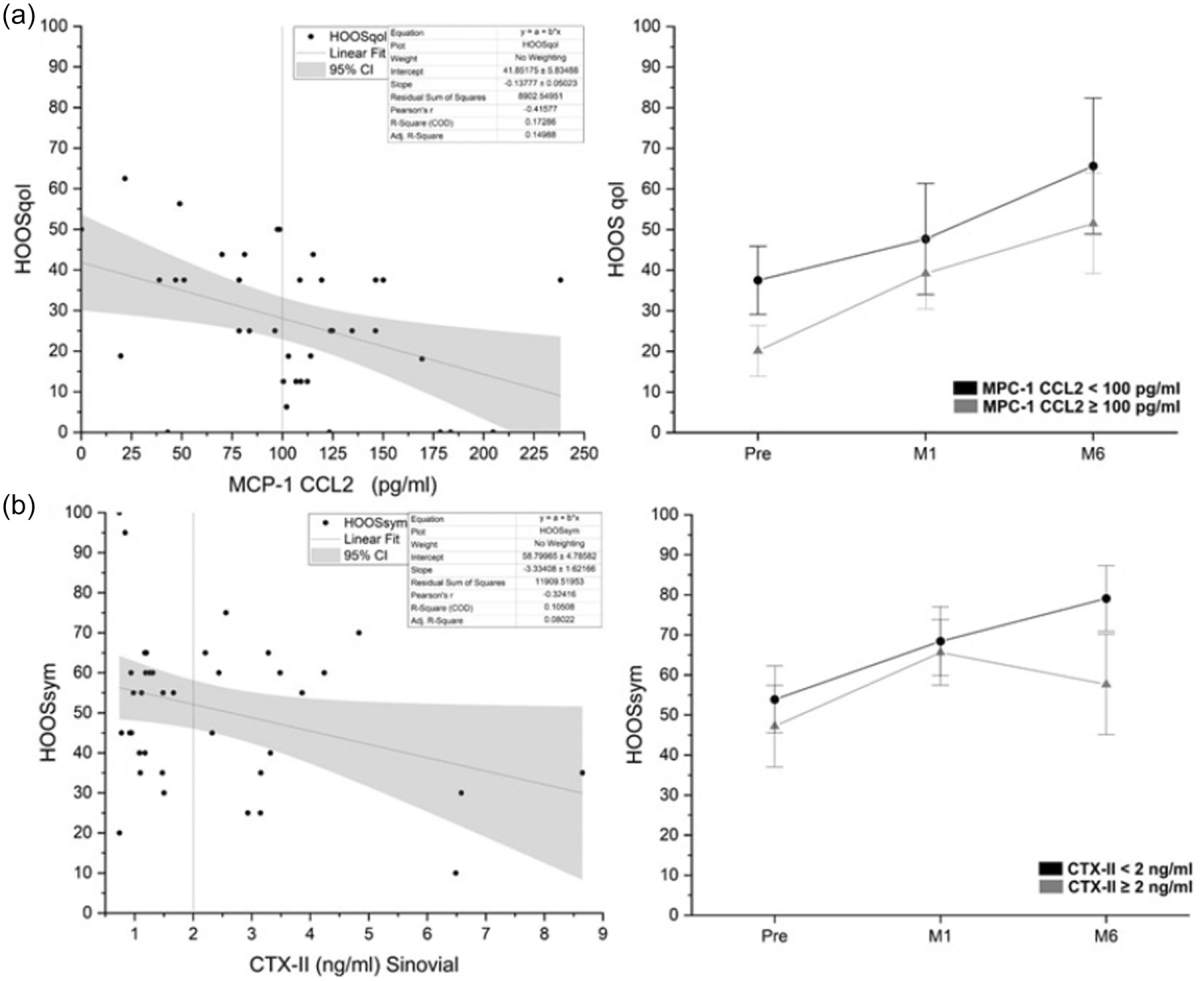
(a) On the right, scatter plots of MCP‐1 CCL2 versus the HOOS QoL subscale at the baseline (pretreatment), and on the left, the effect of the over time of the chosen threshold. (b) On the right, scatter plots of CTX‐II versus the HOOS sym subscale at the baseline (pretreatment), and on the left, the effect of the over time of the chosen threshold. CTX‐II, C‐telopeptide fragments of type II collagen.

CCL2/MCP‐1 resulted to be inversely correlated with HOOS QOL (*r* = −0.42), the scatter plot within the linear regression is reported in Figure 5a. Based on the scatterplot we imposed a threshold at CCL2/MCP‐1 = 100 pg/mL. Based on this threshold, the data were divided to observe (over time) the variation of patients above or below that limit. Also, in this case, patients with a value of CCL2/MCP‐1 above the threshold tend to have lower scores over time.

Only the CTX‐II detected in SFs resulted to be inversely correlated with HOOS sym (*r* = −0.32), the scatterplot is shown in Figure 5b also in this case the HOOS values were at the baseline, the threshold chosen was CTX‐II_synovial = 2 ng/mL. Patients with the CTX‐II_synovial marker above 2 ng/mL tend to have lower HOOS symptoms over time. The presence of CTX‐II in the urine was very low, and it did not show any correlation with clinical data.

COMP was detected in the urine of only 6 patients out of 49 with a concentration near the lower limit of detectability and in the SFs of 10 patients out of 49; therefore, it was excluded from the analysis.

## DISCUSSION

The most important finding of this study was that the use of hydrolyzed collagen treatments was safe, well‐tolerated and did not induce side‐effects and the outcomes were comparable to cortisone and local anaesthetic. The peptide treatment compared to cortisone was less effective, albeit no significant difference was found at 1 month in terms of HOOS and VAS score. However, these reached the same values at 6 months.

Hip arthroscopy is nowadays the most common surgical treatment carried out in patients with FAI. Due to its relatively recent spreading, there are no universally accepted post‐operative management protocols to guide surgeons. Intra‐articular injections at the end of the procedure are widespread, but there is no consensus about what substance is commonly used, and it is not known if an injection has any beneficial effect on the outcome or if any substances work better than others. Post‐operative pain relief, anti‐inflammatory effect and hip function are the goals [7, 8, 13]. Therefore, the research of alternative and effective options as hydrolyzed collagen, successfully already used in treating knee OA [9, 22, 28], is extremely important.

Moreover, in our study, we confirmed that the worse post‐operative outcomes in terms of functional activity and symptoms were found in older and obese patients and in case of acetabular microfracture. These findings are in line with other studies in the literature [14, 17]. Microfractures are carried out in case of severe cartilage damage; therefore, it is not surprising to find worse functional outcomes in these patients when compared to patients with no cartilage lesions. Worst outcomes were also found in case of femoroplasty. The chronic articular damage due to femoral bump in Cam‐type FAI could explain this fact, considering that femoroplasty is carried out only in this case and not in Pincer‐type FAI or traumatic labral tear.

As FAI is involved in the early development of HOA, which is often associated with low‐grade synovitis and synovial inflammation [21], we also investigate the presence of inflammatory molecules in the SF collected at the time of surgery. Among the molecules selected for the analysis, IL‐8 showed an inverse correlation with preoperative total HOOS and symptoms and sport subscales, higher IL‐8 synovial concentration correlated with poorer conditions. The same inverse correlation was observed for CCL2/MCP‐1 only for HOOS QOL subscale. Interestingly, chemokines as IL‐8 and the monocyte chemoattractant protein‐1 (CCL2/MCP‐1), can attract additional inflammatory cells potentiating the inflammatory state in the joint.

Several studies can be found in the literature dealing with some SF biomarkers' presence, and our results are substantially comparable. The concentration of inflammatory biomarkers is high in hip arthropathies and higher in severe OA rather than in mild ones [1]. Matrix degradation markers have been shown to be elevated in patients with late OA and are correlated with radiographic severity [22]. We confirmed the presence of those markers in SFs of FAI patients, but as also shown by Luo et al. [6, 18] we did not find a detectable amount of CTX‐II or COMP in urine samples.

Our study presents potential limitations in the study design and in SF biomarkers detection. First, the absence of a third group of patients where no injections were carried out after the arthroscopy. Over time both groups improved in terms of function and pain, even if we cannot confirm that the effects are related to the injected substances or to the surgical procedure itself.

Moreover, to measure biomarkers in biological fluids, specific and sensitive ELISAs are well‐suited to perform the analysis of one single biomarker at a time. This technique is time‐consuming and expensive for multi‐parameter analyses. On the contrary, multiplex bead‐based flow cytometry offers the opportunity to estimate different markers of interest in the same sample, but the analytes might also present different concentrations in the biological fluids, making it difficult to predict detection for all of them in the same sample in any case.

## CONCLUSIONS

The present study revealed that the use of hydrolyzed collagen treatments is safe, well‐tolerated and comparable to cortisone and local anaesthetics. We did not see any side effects of PEPTYS in our study, but larger‐scale studies are needed to possibly detect less frequent side effects.

Therefore, PEPTYS might be suggested as a good option in post‐operative hip arthroscopy injections.

## AUTHOR CONTRIBUTIONS

**Enrico Tassinari**: Substantial contributions to the conception and design of the work; acquisition, analysis and interpretation of data; patient selection and performing arthroscopy procedure and sample collection. Drafting the work and revising it critically for important intellectual content. Final approval of the version to be published. Agreement to be accountable for all aspects of the work in ensuring that questions related to the accuracy and integrity of any part of the work are appropriately investigated and resolved. **Andrea Minerba**: Acquisition, analysis and interpretation of data; sample collection. Drafting the work and revising it critically for important intellectual content. Final approval of the version to be published. **Tommaso Basile**: Acquisition, analysis and interpretation of data; sample collection. Drafting the work and revising it critically for important intellectual content. Final approval of the version to be published. **Alessio Bucciarelli**: Statistical analysis and interpretation of data. Drafting the work and revising it critically for important intellectual content. Final approval of the version to be published. **Francesco Traina**: Drafting the work and revising it critically for important intellectual content. Final approval of the version to be published. Agreement to be accountable for all aspects of the work in ensuring that questions related to the accuracy and integrity of any part of the work are appropriately investigated and resolved. Final approval of the version to be published. **Brunella Grigolo**: Drafting the work and revising it critically for important intellectual content. Final approval of the version to be published. Agreement to be accountable for all aspects of the work in ensuring that questions related to the accuracy and integrity of any part of the work are appropriately investigated and resolved. **Stefano Zaffagnini**: Drafting the work and revising it critically for important intellectual content. Final approval of the version to be published. Agreement to be accountable for all aspects of the work in ensuring that questions related to the accuracy and integrity of any part of the work are appropriately investigated and resolved. **Eleonora Olivotto**: Substantial contributions to the conception and design of the work; preparation documents for IRB approval; acquisition, analysis and interpretation of data; sample collection. Synovial Fluids analysis. Drafting the work and revising it critically for important intellectual content. Final approval of the version to be published. Agreement to be accountable for all aspects of the work in ensuring that questions related to the accuracy and integrity of any part of the work are appropriately investigated and resolved.

## CONFLICT OF INTEREST STATEMENT

The authors declare no conflicts of interest.

## ETHICS STATEMENT

The study was approved by the ‘Comitato Etico di Area Vasta Emilia Centro della Regione Emilia‐Romagna (CE‐AVEC)’ Prot. gen. 0008219. All patients enroled provided written informed consent.

## Supporting information

Supplementary information.

## Data Availability

The data that support the findings of this study are available from the Last author, [EO], upon reasonable request.
