# Supplementary material for: Post‐operative injection of hydrolyzed collagen peptides shows anti‐inflammatory effect in patients with femoroacetabular impingement improving the early recovery
Source: J Exp Orthop. 2025 Jan 31;12(1):e70158. doi: 10.1002/jeo2.70158 (PMC11783230; doi:10.1002/jeo2.70158)
Supplement: Supplementary file 1 — Supplementary information. [file JEO2-12-e70158-s001.docx]

| **Supplementary Table 1. Equations used for the statistical analysis** | |
| --- | --- |
| **Application** | **Equation** |
| Kit standard curves (Four parameters logistic) | $OD=A_{2}-\frac{A_{1}-A_{2}}{1+\left( \frac{C}{C_{0}} \right)^{p}}$ |
| Kit standard curves (unknown concentration calculation) | $C=C_{0}\left[ \frac{A_{1}-A_{2}}{OD-A_{2}}-1 \right]^{\frac{1}{p}}$ |
| Exclusion of the differences in the HOOS pre intervention between treatments (cortisone vs peptys) | ${HOOS}_{CORR_{t1}}={HOOS_{t1}+(HOOS}_{PRE_{Cort}}-{HOOS}_{PRE_{Pept}})$ |
| Exclusion of the differences in the VAS pre intervention between treatments (cortisone vs peptys) | ${VAS}_{CORR_{t1}}={{VAS}_{t1}+(VAS}_{PRE_{Cort}}-{VAS}_{PRE_{Pept}})$ |
| Normalization for HOOS values | $HOOS_{NORM_{t1}}=\frac{HOOS_{CORR_{t1}}-Min(HOOS_{CORR_{t1}})}{Max\left( HOOS_{CORR_{t1}} \right)-Min(HOOS_{CORR_{t1}})}*100$ |
| Normalization for VAS values | $VAS_{NORM_{t1}}=\frac{VAS_{CORR_{t1}}-Min(VAS_{CORR_{t1}})}{Max\left( VAS_{CORR_{t1}} \right)-Min(VAS_{CORR_{t1}})}*100$ |

**Supplementary Table 2:** Šídák's multiple comparison test on HOOS tot normalized data.

| **Cortisone vs. Peptides** | | | | | | | | |
| --- | --- | --- | --- | --- | --- | --- | --- | --- |
| **Months** | **Mean Diff.** | **95% CI** | **Below threshold?** | **Sig.** | **Adjusted P Value** | |  |  |
| **0** | 0 | -12.45 to 12.45 | No | ns | >0.9999 |  |  |  |
| **1** | 11.51 | -1.163 to 24.19 | No | ns | 0.0847 |  |  |  |
| **6** | 1.012 | -15.52 to 17.54 | No | ns | 0.9983 |  |  |  |
| **Test Details** | | | | | | | | |
| **Months** | **Mean 1** | **Mean 2** | **Mean Diff.** | **SE of diff.** | **N1** | **N2** | **T** | **DF** |
| **0** | 57.68 | 57.68 | 0 | 5.024 | 24 | 24 | 0 | 46 |
| **1** | 80.63 | 69.12 | 11.51 | 5.082 | 24 | 22 | 2.266 | 39.01 |
| **6** | 80.69 | 79.68 | 1.012 | 6.644 | 23 | 23 | 0.1523 | 41.43 |

**Supplementary Table 3:** Šídák's multiple comparison test on HOOS sym normalized data.

| **Cortisone vs. Peptides** | | | | | | | | |
| --- | --- | --- | --- | --- | --- | --- | --- | --- |
| **Months** | **Mean Diff.** | **95% CI** | **Below threshold?** | **Sig.** | **Adjusted P Value** | |  |  |
| **0** | 0 | -13.83 to 13.82 | No | ns | >0.9999 |  |  |  |
| **1** | 8.16 | -5.189 to 21.50 | No | ns | 0.3552 |  |  |  |
| **6** | -1.63 | -18.66 to 15.39 | No | ns | 0.9934 |  |  |  |
| **Test Details** | | | | | | | | |
| **Months** | **Mean 1** | **Mean 2** | **Mean Diff.** | **SE of diff.** | **N1** | **N2** | **T** | **DF** |
| **0** | 56.3 | 56.3 | -0.00435 | 5.573 | 24 | 23 | 0.00078 | 44.9 |
| **1** | 75.88 | 67.73 | 8.156 | 5.367 | 24 | 22 | 1.52 | 42.04 |
| **6** | 73.37 | 75 | -1.635 | 6.858 | 23 | 23 | 0.2384 | 43.72 |

**Supplementary Table 4:** Šídák's multiple comparison test on HOOS pain normalized data.

| **Cortisone vs. Peptides** | | | | | | | | |
| --- | --- | --- | --- | --- | --- | --- | --- | --- |
| **Months** | **Mean Diff.** | **95% CI** | **Below threshold?** | **Sig.** | **Adjusted P Value** | |  |  |
| **0** | 0 | -15.45 to 15.45 | No | ns | >0.9999 |  |  |  |
| **1** | 11.07 | -1.219 to 23.36 | No | ns | 0.0884 |  |  |  |
| **6** | 1.495 | -15.24 to 18.23 | No | ns | 0.9947 |  |  |  |
| **Test Details** | | | | | | | | |
| **Months** | **Mean 1** | **Mean 2** | **Mean Diff.** | **SE of diff.** | **N1** | **N2** | **T** | **DF** |
| **0** | 59.35 | 59.35 | -0.00033 | 6.222 | 24 | 23 | 5.24E-05 | 43.72 |
| **1** | 86.64 | 75.57 | 11.07 | 4.933 | 24 | 22 | 2.244 | 40.17 |
| **6** | 83.78 | 82.28 | 1.495 | 6.735 | 23 | 23 | 0.2219 | 42.95 |

**Supplementary Table 5:** Šídák's multiple comparison test on HOOS funct normalized data.

| **Cortisone vs. Peptides** | | | | | | | | |
| --- | --- | --- | --- | --- | --- | --- | --- | --- |
| **Months** | **Mean Diff.** | **95% CI** | **Below threshold?** | **Sig.** | **Adjusted P Value** | |  |  |
| **0** | 0 | -16.50 to 16.51 | No | ns | >0.9999 |  |  |  |
| **1** | 12.28 | -1.177 to 25.74 | No | ns | 0.0829 |  |  |  |
| **6** | 4.227 | -12.55 to 21.01 | No | ns | 0.8988 |  |  |  |
| **Test Details** | | | | | | | | |
| **Months** | **Mean 1** | **Mean 2** | **Mean Diff.** | **SE of diff.** | **N1** | **N2** | **T** | **DF** |
| **0** | 65.21 | 65.2 | 0.001087 | 6.647 | 24 | 23 | 0.000164 | 43.5 |
| **1** | 87.21 | 74.93 | 12.28 | 5.404 | 24 | 22 | 2.273 | 40.55 |
| **6** | 88.76 | 84.53 | 4.227 | 6.737 | 23 | 23 | 0.6274 | 40.56 |

**Supplementary Table 6:** Šídák's multiple comparison test on HOOS sport normalized data.

| **Cortisone vs. Peptides** | | | | | | | | |
| --- | --- | --- | --- | --- | --- | --- | --- | --- |
| **Months** | **Mean Diff.** | **95% CI** | **Below threshold?** | **Sig.** | **Adjusted P Value** | |  |  |
| **0** | 0 | -17.53 to 17.53 | No | ns | >0.9999 |  |  |  |
| **1** | 22.23 | 5.216 to 39.25 | Yes | ** | 0.007 |  |  |  |
| **6** | 2.426 | -17.84 to 22.70 | No | ns | 0.9874 |  |  |  |
| **Test Details** | | | | | | | | |
| **Months** | **Mean 1** | **Mean 2** | **Mean Diff.** | **SE of diff.** | **N1** | **N2** | **T** | **DF** |
| **0** | 48.38 | 48.38 | -0.00062 | 7.059 | 24 | 23 | 8.73E-05 | 43.15 |
| **1** | 75.29 | 53.06 | 22.23 | 6.807 | 24 | 22 | 3.266 | 37.18 |
| **6** | 74.99 | 72.57 | 2.426 | 8.154 | 23 | 23 | 0.2975 | 42.43 |

**Supplementary Table 7:** Šídák's multiple comparison test on HOOS qol normalized data.

| **Cortisone vs. Peptides** | | | | | | | | |
| --- | --- | --- | --- | --- | --- | --- | --- | --- |
| **Months** | **Mean Diff.** | **95% CI** | **Below threshold?** | **Sig.** | **Adjusted P Value** | |  |  |
| **0** | 0 | -12.28 to 12.28 | No | ns | >0.9999 |  |  |  |
| **1** | 3.008 | -14.07 to 20.08 | No | ns | 0.9612 |  |  |  |
| **6** | -11.8 | -32.35 to 8.741 | No | ns | 0.4091 |  |  |  |
| **Test Details** | | | | | | | | |
| **Months** | **Mean 1** | **Mean 2** | **Mean Diff.** | **SE of diff.** | **N1** | **N2** | **T** | **DF** |
| **0** | 32.62 | 32.62 | -0.00156 | 4.948 | 24 | 23 | 0.000315 | 44.37 |
| **1** | 49.05 | 46.04 | 3.008 | 6.809 | 24 | 22 | 0.4418 | 34.94 |
| **6** | 53.7 | 65.5 | -11.8 | 8.275 | 23 | 23 | 1.426 | 43.71 |

**Supplementary Table 8:** Šídák's multiple comparison test on VAS normalized data.

| **Cortisone vs. Peptides** | | | | | | | | |
| --- | --- | --- | --- | --- | --- | --- | --- | --- |
| **Months** | **Mean Diff.** | **95% CI** | **Below threshold?** | **Sig.** | **Adjusted P Value** | |  |  |
| **0** | 0 | -1.975 to 1.980 | No | ns | >0.9999 |  |  |  |
| **1** | -0.8614 | -2.995 to 1.272 | No | ns | 0.6788 |  |  |  |
| **6** | -0.9725 | -3.722 to 1.777 | No | ns | 0.7554 |  |  |  |
| **Test Details** | | | | | | | | |
| **Months** | **Mean 1** | **Mean 2** | **Mean Diff.** | **SE of diff.** | **N1** | **N2** | **T** | **DF** |
| **0** | 6.043 | 6.041 | 0.002526 | 0.7924 | 23 | 21 | 0.003188 | 38.55 |
| **1** | 4.188 | 5.049 | -0.8614 | 0.843 | 16 | 18 | 1.022 | 29.55 |
| **6** | 3.5 | 4.473 | -0.9725 | 1.076 | 14 | 16 | 0.9038 | 25.39 |

| **Supplementary Table 9:** General Linear Regression to determine which variables are significant for the HOOS and its subscales.  (The HOOS was corrected at the baseline and normalized). | | | | | | | | | | | | | | | | | | | | | | | | |
| --- | --- | --- | --- | --- | --- | --- | --- | --- | --- | --- | --- | --- | --- | --- | --- | --- | --- | --- | --- | --- | --- | --- | --- | --- |
|  | **Total** | | | | **Symptoms** | | | | **Pain** | | | | **Function** | | | | **Sport** | | | | **Quality** | | | |
|  | **B** | **Sig.** | **95% CI L** | **95% CI H** | **B** | **Sig.** | **95% CI L** | **95% CI H** | **B** | **Sig.** | **95% CI L** | **95% CI H** | **B** | **Sig.** | **95% CI L** | **95% CI H** | **B** | **Sig.** | **95% CI L** | **95% CI H** | **B** | **Sig.** | **95% CI L** | **95% CI H** |
| **Sex=0** | -7.32 | 0.080 | -15.52 | 0.88 | **-13.19** | **0.002** | **-21.60** | **-4.78** | -8.12 | 0.057 | -16.49 | 0.26 | -7.01 | 0.110 | -15.61 | 1.60 | -8.33 | 0.104 | -18.39 | 1.73 | 1.59 | 0.755 | -8.51 | 11.69 |
| **Side=0** | -0.54 | 0.889 | -8.12 | 7.05 | -2.67 | 0.501 | -10.49 | 5.15 | 0.14 | 0.974 | -8.16 | 8.44 | 1.43 | 0.720 | -6.44 | 9.30 | -5.96 | 0.204 | -15.18 | 3.27 | -2.40 | 0.622 | -12.02 | 7.21 |
| **A.Chond.≥2** | -3.96 | 0.572 | -17.80 | 9.87 | 3.56 | 0.593 | -9.60 | 16.72 | -4.47 | 0.525 | -18.35 | 9.42 | -4.30 | 0.572 | -19.30 | 10.71 | -8.28 | 0.316 | -24.58 | 8.01 | -6.58 | 0.394 | -21.82 | 8.66 |
| **F.Chond.≥2** | 6.08 | 0.299 | -5.45 | 17.61 | -0.20 | 0.971 | -10.90 | 10.51 | 7.24 | 0.243 | -4.98 | 19.45 | 6.49 | 0.291 | -5.63 | 18.61 | 11.26 | 0.094 | -1.97 | 24.49 | 4.14 | 0.533 | -8.98 | 17.26 |
| **L.Sut.=0** | 1.23 | 0.767 | -6.97 | 9.43 | -2.82 | 0.517 | -11.40 | 5.77 | 2.73 | 0.497 | -5.22 | 10.69 | 1.62 | 0.712 | -7.04 | 10.27 | -2.26 | 0.661 | -12.45 | 7.93 | 4.45 | 0.360 | -5.15 | 14.05 |
| **L.Shav.=0** | 1.42 | 0.768 | -8.09 | 10.94 | -0.39 | 0.940 | -10.52 | 9.75 | 2.56 | 0.581 | -6.59 | 11.70 | 3.00 | 0.548 | -6.88 | 12.89 | -3.14 | 0.580 | -14.34 | 8.06 | -1.15 | 0.844 | -12.73 | 10.43 |
| **MicroFra=0** | 12.84 | 0.049 | 0.08 | 25.61 | **14.45** | **0.013** | **3.10** | **25.79** | 9.62 | 0.136 | -3.08 | 22.32 | 11.39 | 0.155 | -4.36 | 27.13 | **20.88** | **0.010** | **5.07** | **36.69** | **17.08** | **0.013** | **3.72** | **30.44** |
| **A.Plasty=0** | 1.96 | 0.670 | -7.13 | 11.06 | -0.20 | 0.963 | -8.59 | 8.19 | 3.31 | 0.487 | -6.10 | 12.73 | 0.41 | 0.935 | -9.50 | 10.32 | 0.06 | 0.992 | -12.06 | 12.19 | 9.84 | 0.070 | -0.82 | 20.50 |
| **F.Plasty=0** | 10.37 | 0.065 | -0.65 | 21.38 | **14.27** | **0.005** | **4.50** | **24.04** | **13.49** | **0.038** | **0.75** | **26.23** | 5.86 | 0.307 | -5.46 | 17.19 | 11.25 | 0.127 | -3.25 | 25.75 | **15.83** | **0.041** | **0.65** | **31.01** |
| **Tonnis≥1** | -2.62 | 0.558 | -11.47 | 6.22 | -4.28 | 0.358 | -13.46 | 4.91 | -2.34 | 0.606 | -11.31 | 6.63 | -1.91 | 0.682 | -11.09 | 7.28 | -2.43 | 0.671 | -13.74 | 8.88 | -4.32 | 0.432 | -15.15 | 6.52 |
| **Age** | **-0.40** | **0.049** | **-0.81** | **0.00** | -0.10 | 0.584 | -0.48 | 0.27 | -0.36 | 0.077 | -0.77 | 0.04 | **-0.49** | **0.029** | **-0.93** | **-0.05** | **-0.50** | **0.041** | **-0.98** | **-0.02** | -0.42 | 0.073 | -0.88 | 0.04 |
| **BMI** | **-1.11** | **0.022** | **-2.05** | **-0.17** | **-0.98** | **0.038** | **-1.90** | **-0.06** | **-1.19** | **0.015** | **-2.14** | **-0.23** | **-1.16** | **0.021** | **-2.15** | **-0.18** | **-1.69** | **0.006** | **-2.88** | **-0.50** | -0.25 | 0.657 | -1.37 | 0.86 |

All the follow-ups (1-6 months) were inserted in the model, considering the time as a continuous variable(time was not shown in the table because always significant). The significant terms are reported in bold. (Sex=0=female; Side=0=left, A.Chond.= Acetabular Chondropathy; F.Chond.= Femoral Chondropathy; L.Sut= labral suture; L.Shav.= labral shaving; MicroFra= microfractures; A.Plasty.= Acetabuloplasty; F.Plasty= Femoroplasty; 0 means absence of the variable; BMI= body mass index).

| **Supplementary Table 10:** General Linear Regression to determine which variables are significant for VAS. (VAS were corrected at the baseline and normalized). The timepoints were insert in the model as a continuous variable and not shown in the table because always significant). | | | | |
| --- | --- | --- | --- | --- |
|  | **B** | **Sig.** | **95% CI L** | **95% CI H** |
| **Intercept** | 4.68 | 0.318 | -4.57 | 13.92 |
| **Treat=0** | -0.71 | 0.314 | -2.11 | 0.69 |
| **Sex=0** | 1.15 | 0.207 | -0.65 | 2.96 |
| **Side=0** | 0.10 | 0.877 | -1.23 | 1.43 |
| **A.Chond.≥2** | -0.72 | 0.531 | -2.98 | 1.55 |
| **F.Chond.≥2** | 0.34 | 0.756 | -1.84 | 2.52 |
| **L.Sut.=0** | -0.64 | 0.393 | -2.13 | 0.85 |
| **L.Shav.=0** | -0.19 | 0.830 | -1.89 | 1.52 |
| **MicroFra=0** | 0.65 | 0.700 | -2.71 | 4.02 |
| **A.Plasty=0** | -1.21 | 0.082 | -2.57 | 0.16 |
| **F.Plasty=0** | 0.09 | 0.975 | -5.43 | 5.60 |
| **Tonnis≥1** | 0.01 | 0.993 | -1.59 | 1.60 |
| **Age** | 0.03 | 0.517 | -0.05 | 0.10 |
| **BMI** | 0.01 | 0.944 | -0.25 | 0.27 |
| **Time** | -0.21 | 0.059 | -0.42 | 0.01 |

*
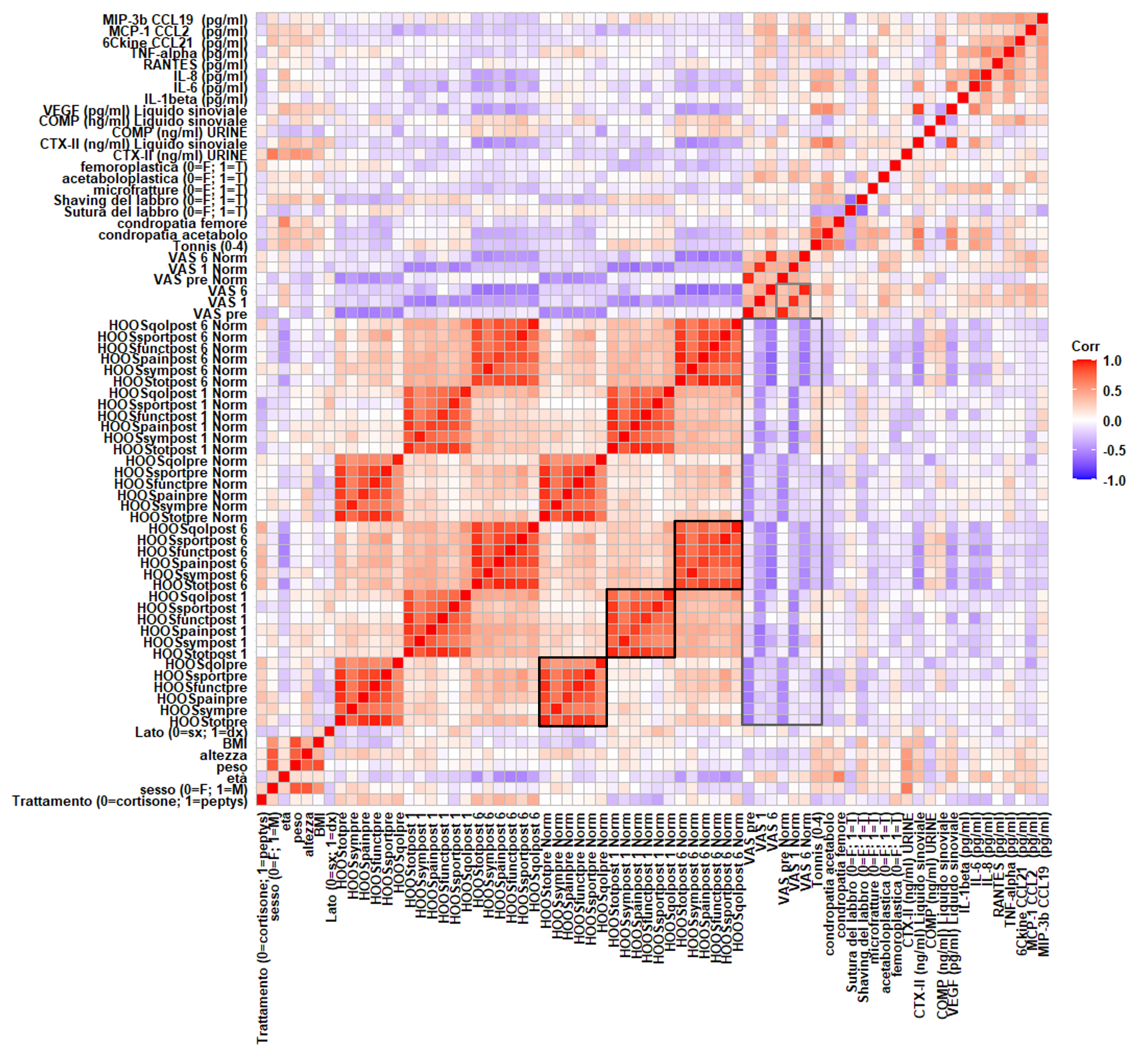
*

**Supplementary Figure 1:** Spearman correlation matrix to perform an explorative data analysis. The highlighted regions are of particular interest. The normalized HOOS maintain the same information of the bare HOOS, in fact a strong direct correlation among them is present (black rectangles). The same conclusion can be deduced by observing the correlation among the bare and the normalized VAS (light grey rectangle). The normalization preserves the information between VAS and HOOS (mid grey rectangle).

*
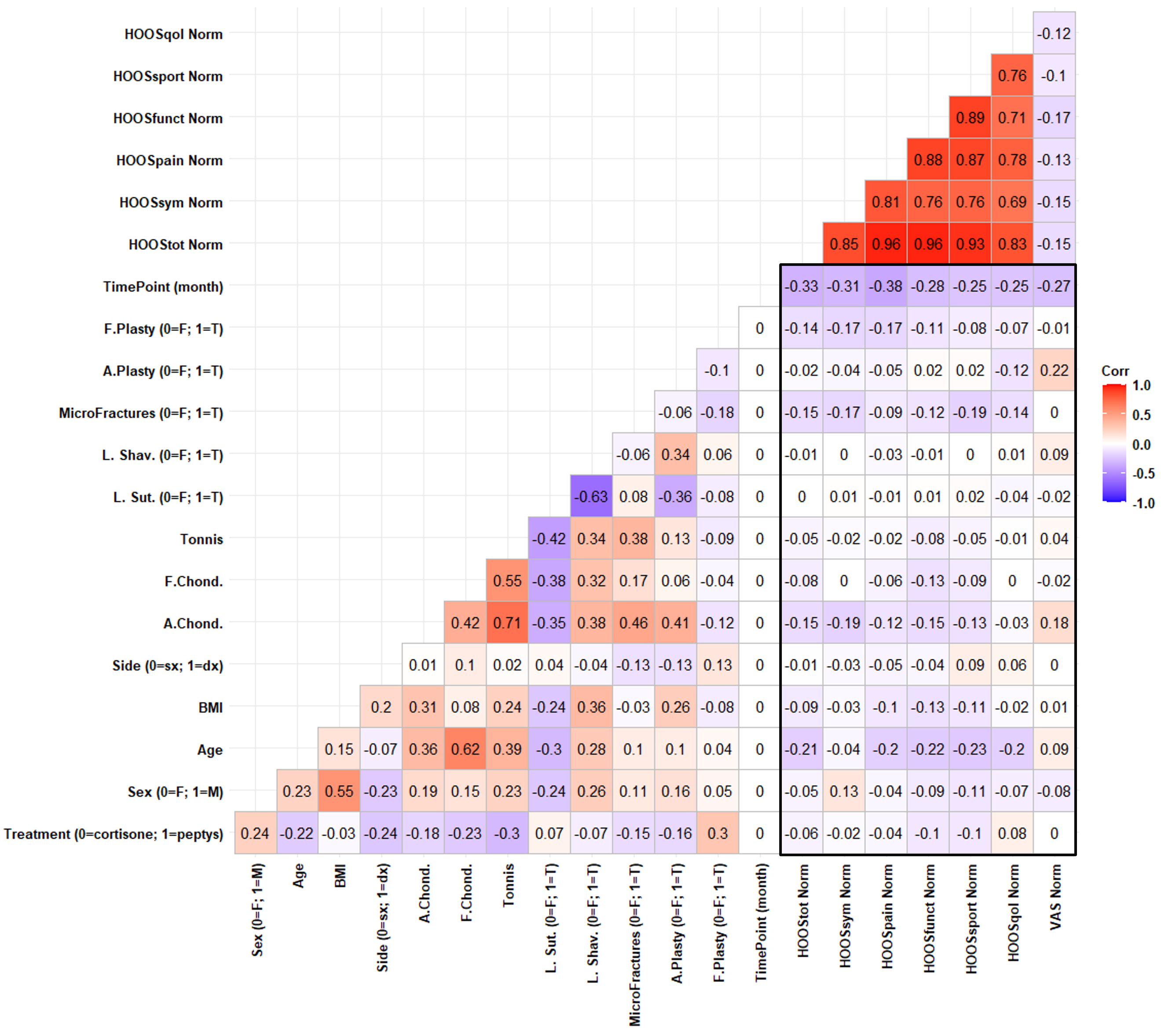
*

**Supplementary Figure 2**: Spearman correlation matrix linking the clinical data to HOOS and VAS. In this case we use the normalized HOOS and VAS based on the HOOS and VAS collected at Time 0 (before the treatment application). In a first analysis the Treatment result to be not influential on the HOOS and VAS scale, meaning that the two treatments may be considered as interchangeable. From an initial analysis HOOS and VAS were slightly correlated to the Timepoint. It should be noticed that the correlation is slightly negative, and this is due the presence of few outliers.


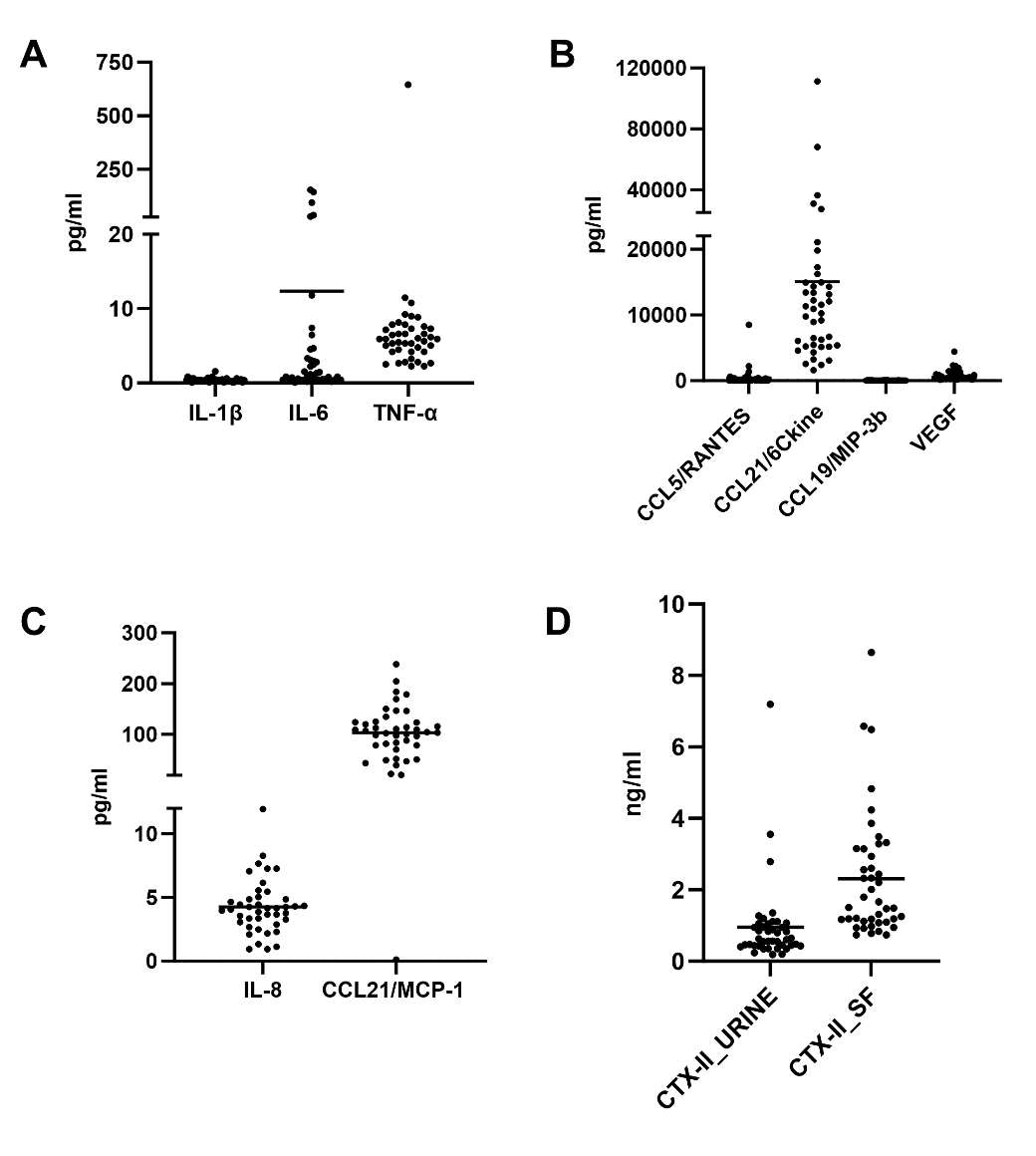


**Supplementary Figure 3:** Biomarkers analysis. Cytokines (panel A) and chemokines (B and C) release in synovial fluid of FAI patients. C-telopeptide fragments of type II collagen (CTX-II) in synovial fluid and urine of FAI patients (panel D).

**
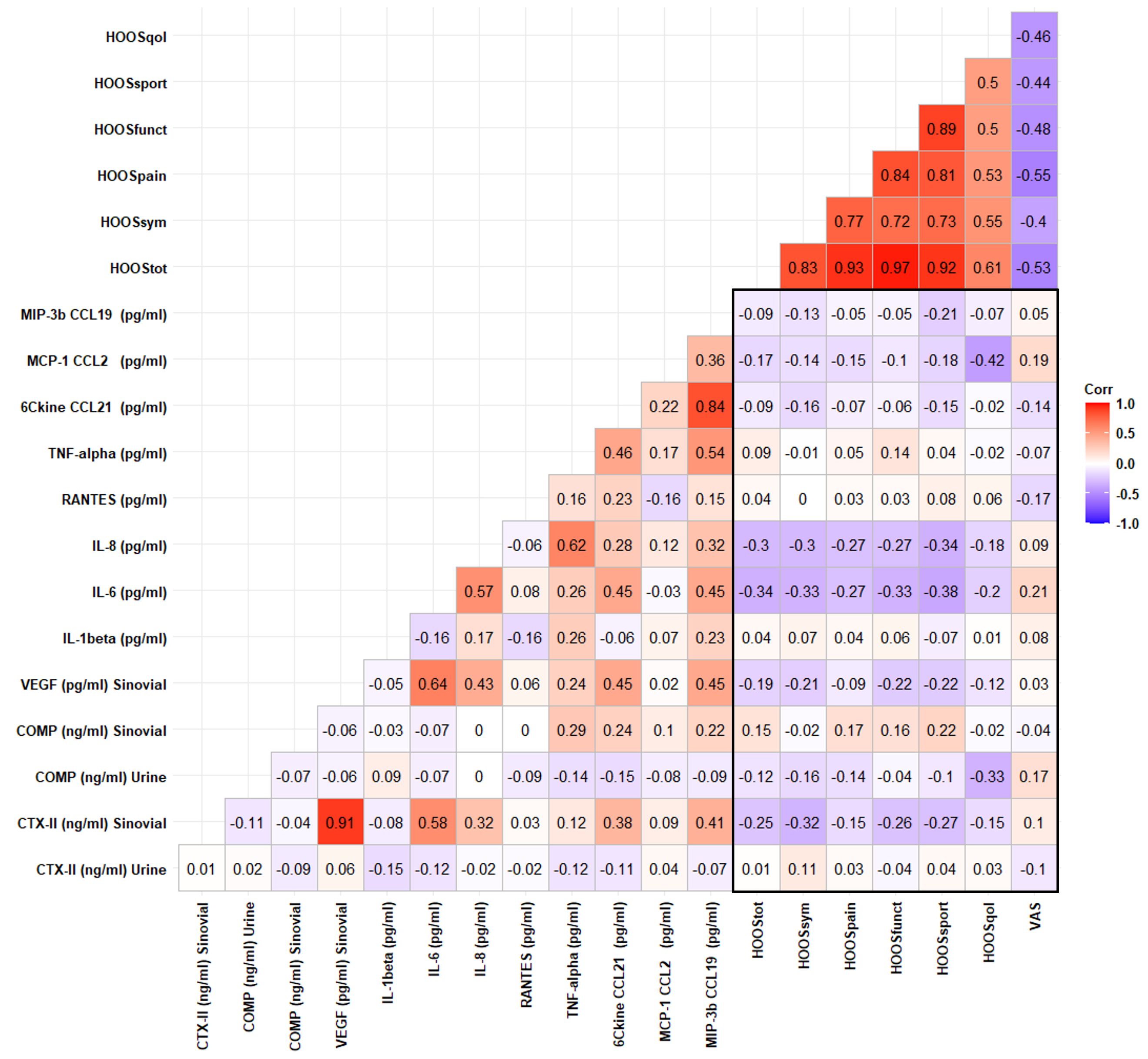
**

**Supplementary Figure 4:** Pearsons Correlation Matrix linking the Biological Markers and HOOS and VAS all collected pre-intervention. The correlation in which we are interested are enclosed in the rectangle.
